# The Dental Operating Microscope in Endodontics: A Narrative Review of Clinical Applications, Supporting Evidence, and Limitations

**DOI:** 10.7759/cureus.117039

**Published:** 2026-09-27

**Authors:** Serkan Ucmak

**Affiliations:** 1 Department of Endodontics, Faculty of Dentistry, Selçuk University, Konya, TUR

**Keywords:** dental operating microscope, endodontic microsurgery, endodontics, magnification, regenerative endodontics, root canal treatment, second mesiobuccal canal, vital pulp therapy

## Abstract

The dental operating microscope (DOM) is now widely used in endodontics, but the strength of the evidence supporting its use differs between procedures. This narrative review summarizes the historical development and technical principles of the DOM, evaluates the evidence for its use in the main endodontic procedures, and outlines its limitations. A structured PubMed search covering 1981 to 2026 was supplemented by hand-searching of reference lists. The DOM combines magnification, coaxial illumination, and an upright working posture. In non-surgical root canal treatment, magnification consistently improves the detection of canal orifices, particularly the second mesiobuccal (MB2) canal of maxillary molars, and supports the management of calcified canals, complex anatomy, separated instruments, and perforations. Direct outcome data in non-surgical treatment are limited to observational studies. In vital pulp therapy and regenerative endodontic procedures, the DOM is used mainly to assess the pulp tissue and bleeding and to place materials precisely, but its independent effect on outcomes has not been tested. The strongest outcome evidence comes from endodontic microsurgery, in which a meta-analysis reported a pooled success rate of 94% with high-power magnification, compared with 88% without it, with the benefit concentrated in molars. However, two Cochrane reviews found no eligible randomized controlled trials, and a retrospective comparison of loupes and the microscope in endodontic surgery found no significant difference between them. Longer treatment time, cost, the learning curve, and restricted access in some patients are the main practical limitations. The DOM proves most advantageous when managing microscopic features that require high magnification and significantly impact the clinical outcome. Well-designed clinical trials are needed to determine whether its diagnostic advantages lead to better long-term outcomes.

## Introduction and background

Apical periodontitis is an inflammatory disease of the periradicular tissues that is caused primarily by microbial infection of the root canal system [1,2]. The microorganisms involved are organized as biofilms embedded in an extracellular polymeric matrix, and as biofilms mature, they become more resistant to mechanical removal and chemical disinfection [2-4]. The fundamental goals of root canal treatment are therefore to remove infected and necrotic tissue, to disinfect the root canal system as thoroughly as possible, to seal it against reinfection, and to preserve the tooth [2,5]. When the pulp is still vital, or when root development is incomplete, the aim shifts toward preserving or regenerating pulp tissue [6,7].

All of these goals depend on what the clinician can see. Root canal anatomy is complex: orifices may be narrow, calcified, or covered by dentin; additional canals and roots are common; and cracks, perforations, and resorptive defects may be only fractions of a millimeter wide [8-10]. A canal that is not located cannot be cleaned, and cone-beam computed tomography (CBCT) studies have shown that endodontically treated teeth with untreated canals are significantly more likely to have apical periodontitis [11,12]. At a normal working distance, the unaided eye often cannot resolve these structures. In addition, near visual acuity declines with age, and in practicing dentists, this decline becomes clinically relevant from about 40 years of age [13,14]. Loupes improve near visual acuity, and under simulated clinical conditions, the microscope provides a further improvement even when the magnification factors are similar [13,15].

Visual demands also have ergonomic consequences. Without adequate magnification, clinicians tend to move closer to the operating field by flexing the neck and trunk. Studies using validated posture assessment methods have found that working with the DOM is associated with a more favorable posture than working with loupes or without magnification, both in preclinical dental students and in postgraduate endodontic students [16-18]. On the basis of these visual and ergonomic considerations, an expert consensus of the Chinese Stomatological Association recommends the DOM for better visualization of fine structures, reduced strain on the ciliary muscle, and a more upright working position [19].

Despite its wide use, the evidence base for the DOM is uneven. Its adoption in endodontics was driven substantially by training requirements, and two Cochrane reviews found no randomized controlled trials eligible for inclusion [20-22]. Many descriptions of the microscope list its applications without distinguishing between procedures for which outcome data exist and procedures for which the benefit is assumed. This distinction matters for clinicians, educators, and practices deciding when the additional time and cost of the microscope are justified.

The objectives of this narrative review were (i) to summarize the historical development and technical principles of the DOM that are relevant to clinical use; (ii) to evaluate the evidence for its use in non-surgical root canal treatment, non-surgical retreatment, vital pulp therapy, regenerative endodontic procedures, and endodontic microsurgery; (iii) to summarize its ergonomic and educational aspects and its use in other dental disciplines; and (iv) to identify its limitations and the situations in which its use is less justified.

## Review

Methods of the literature search

A literature search was performed in PubMed (National Center for Biotechnology Information) on 15 September 2026 for records published between January 1, 1981, and the search date. The start date was chosen because the first microscope designed specifically for dentistry was described in 1981 [23]. The main search combined Medical Subject Headings (MeSH) with free-text terms searched in titles and abstracts using the following string: (“Microsurgery”[MeSH] OR “operating microscope*”[tiab] OR “dental microscope*”[tiab] OR “surgical microscope*”[tiab] OR magnification[tiab] OR loupe*[tiab] OR “enhanced visuali*”[tiab]) AND (“Endodontics”[MeSH] OR “Root Canal Therapy”[MeSH] OR “Apicoectomy”[MeSH] OR “Pulpotomy”[MeSH] OR “Dental Pulp Capping”[MeSH] OR endodont*[tiab] OR “root canal*”[tiab] OR mesiobuccal[tiab] OR “calcified canal*”[tiab] OR “pulp canal obliteration”[tiab] OR “separated instrument*”[tiab] OR perforation*[tiab] OR “regenerative endodontic*”[tiab] OR “dynamic navigation”[tiab] OR robot*[tiab] OR “computer-assisted”[tiab]).

Two supplementary searches addressed topics outside endodontic treatment: (“operating microscope*”[tiab] OR magnification[tiab] OR loupe*[tiab]) AND (ergonomic*[tiab] OR posture[tiab] OR “visual acuity”[tiab] OR “Education, Dental”[MeSH]); and (“operating microscope*”[tiab] OR microsurg*[tiab]) AND (restorative[tiab] OR periodont*[tiab] OR implant*[tiab]). The English-language filter was applied. No study design or species filters were used, because ex vivo laboratory studies were relevant to several questions. Records were exported to EndNote 21 (Clarivate Analytics, London, UK), and duplicates were removed. The reference lists of included reviews and key primary studies were hand-searched. No other databases were searched.

Systematic reviews and meta-analyses; randomized and non-randomized clinical studies; cohort, case-control, and cross-sectional studies; ex vivo comparative studies; surveys; and consensus and position statements that addressed magnification or microscope-assisted procedures in dentistry were eligible. Case reports were included only when they illustrated an application for which no comparative studies were available (for example, complex anatomy, calcified canals, or regenerative endodontic procedures) or when they were of historical importance. Studies that did not evaluate or use magnification, conference abstracts, letters without original data, and reports duplicating previously published data were excluded. The author screened the retrieved titles and abstracts and assessed the full texts of potentially relevant records against these criteria; in keeping with the narrative design, the selection prioritized the most informative and highest-level evidence for each clinical question rather than aiming for exhaustive inclusion, and the number of records at each stage was not formally recorded. Further sources were identified by hand-searching reference lists. Background references on the etiology of endodontic disease, treatment principles, and review methodology were cited to provide context but were not part of the evidence synthesis. In total, 119 references are cited. The evidence was organized by clinical procedure, and within each section, higher levels of evidence are presented first. Because this is a narrative review, no formal risk-of-bias assessment or quantitative synthesis was performed. The Scale for the Assessment of Narrative Review Articles (SANRA) was used as a guide for reporting [24].

Historical development

Operating microscopes were used in otolaryngology and ophthalmology from the 1950s and later became standard in neurosurgery and microvascular surgery. In 1981, Apotheker and Jako described the first microscope designed specifically for dentistry [23], and its clinical applications were outlined in the same year [25]. The first commercial model had a fixed magnification and required an uncomfortable working posture; it was not commercially successful, but the concept was retained in later designs [26,27].

Clinical applications then expanded. Reuben and Apotheker described apical surgery under the dental microscope in 1984 [28], and Selden reported its use for the non-surgical treatment of calcified canals in 1989 [29]. In 1993, Pecora and Andreana described a structured technique for microscope-assisted endodontic surgery [30]. Together with the introduction of ultrasonic root-end preparation and microinstruments, this work contributed to the transition from traditional apicoectomy, performed with a bur under direct vision, to modern endodontic microsurgery [31].

Adoption was nevertheless slow, as Selden noted in 2002 [26]. An important step was the decision of the Commission on Dental Accreditation in 1998 to include proficiency in the use of the microscope in the educational standards for postgraduate endodontic programs in the United States [19,26]. In a 2008 survey of members of the American Association of Endodontists, 90% of respondents reported having and using a microscope [20]. Use was reported by 95% of endodontists who had completed training fewer than 10 years earlier, compared with 78% of those who had completed training more than 21 years earlier [20]. This difference is consistent with the effect of the 1998 training requirement, although a survey cannot establish a causal relationship.

Outside the specialty of endodontics, the incorporation of the DOM into clinical practice remains uneven, and recent multicountry survey data show that its use increases with the level of endodontic training [32]. The integration of magnification into undergraduate education also varies. A survey of endodontic teaching leads in all dental schools in the United Kingdom and Ireland found that magnification was inconsistently included in undergraduate curricula; most schools did not expect students to use it, and cost and a lack of trained staff were the main barriers reported [33]. The British Endodontic Society Teachers of Endodontology Group subsequently recommended dental loupes as a minimum requirement for undergraduate non-surgical endodontics [34]. The main milestones are shown in Figure 1.

**Figure 1:**
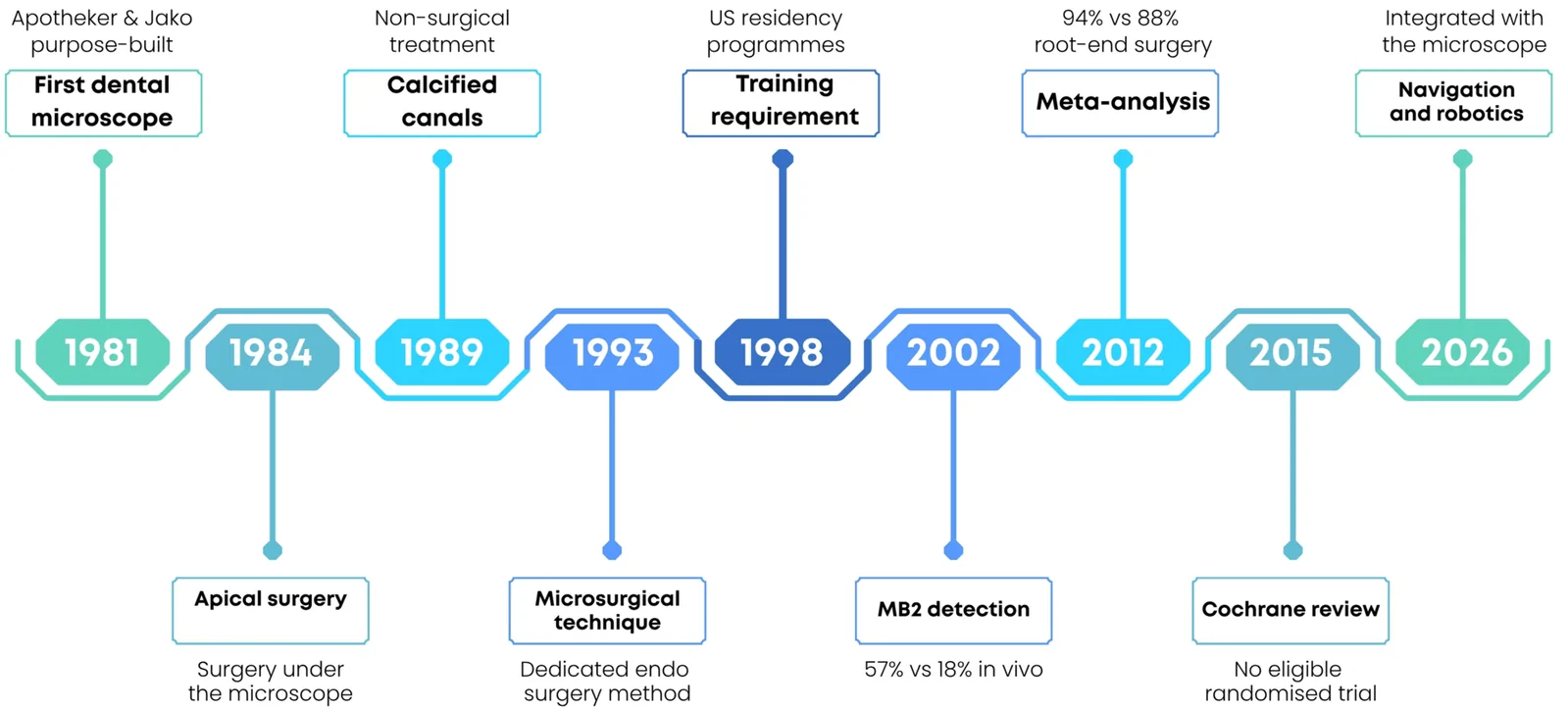
Milestones in the development of the dental operating microscope in endodontics, 1981–2026. Timeline from the first microscope designed for dentistry to the introduction of microsurgical techniques, the inclusion of microscope training in US postgraduate endodontic programs, and the current integration with navigation and robotic systems. Illustration created by the author from the literature cited in the text. MB2: Second mesiobuccal canal

Technical principles

Optical System and Magnification

Total magnification is determined by four factors: the eyepiece magnification, the focal length of the binocular tube, the magnification changer setting, and the focal length of the objective lens. The conventional calculation applies only to microscopes with a fixed-focal-length objective and cannot be used for variable-focal-length designs [19]. The objective focal length also determines the working distance between the microscope and the operating field [19,35].

Three magnification bands are usually distinguished [19,31]. Low magnification (approximately ×3 to ×8) provides a wide, bright field with a large depth of field and is used for orientation. Mid-range magnification (approximately ×9 to ×16) is used for most operative steps. High magnification (above approximately ×16) narrows the field and the depth of field and requires more light; it is used to inspect fine details such as the canal walls, perforation margins, and the resected root surface [19,31]. Magnification is therefore adjusted repeatedly during a procedure rather than set once. Typical ranges for common endodontic procedures are summarized in Table 1.

**Table 1:** Typical magnification ranges for common endodontic procedures with the dental operating microscope Ranges are indicative and are adapted from references [19,27,31]. The settings available depend on the microscope model and eyepiece/objective combination. MB2: Second mesiobuccal canal

Procedure/step	Typical magnification	Purpose
Orientation, rubber dam check, initial access outline; flap design and soft-tissue handling in surgery	Low (×3-×8)	Wide field and large depth of field for orientation
Pulp chamber floor inspection; locating canal orifices (including MB2 and calcified canals); ultrasonic troughing	Mid (×9-×16)	Identification of the floor anatomy, dentine color changes, and orifices
Assessment of exposed pulp and hemostasis (vital pulp therapy); placement of blood clot, matrix, and calcium silicate cement (regenerative procedures)	Low to mid (×6-×12)	Evaluation of tissue and precise material placement
Separated instrument removal (staging platform, ultrasonic loosening); perforation repair	Mid to high (×10-×20)	Visualization of fragment head, perforation margins, and material adaptation
Osteotomy and root-end resection	Low to mid (×4-×12)	Location of the root apex and control of the resection
Inspection of canal walls, cracks, resected root surface (isthmuses, microcracks); root-end preparation and filling check	High (×16-×25)	Detection of fine details with a narrow field and shallow depth of field

The optical advantage of the microscope over loupes has been measured directly. In a phantom-head study under simulated clinical conditions, visual acuity differed significantly between all tested conditions, and the microscope performed better than loupes even at similar magnification factors [15]. Among loupes, Keplerian (prismatic) systems performed better than Galilean systems [36], and the benefit of magnification has been shown in both students and experienced clinicians [37]. However, dentists’ self-reported experience with magnification was poorly related to their measured visual acuity, which suggests that magnification devices should be selected on the basis of objective testing [38]. A comparison of the DOM with a high-resolution videoscope showed that, at three magnification settings, the microscope achieved resolutions of 32, 40.3, and 50.8 line pairs/mm, with depths of field of 15, 10, and 6 mm, respectively; the videoscope achieved 20.1 line pairs/mm with a depth of field of 5 mm [39]. These values illustrate the basic trade-off in microscope use: higher resolution is obtained at the cost of a shallower depth of field. Better visual acuity does not, however, automatically translate into better clinical outcomes; clinical comparisons of the microscope and loupes are discussed in the sections on canal location and endodontic microsurgery [40,41].

Illumination and Filters

The DOM delivers light along the optical axis (coaxial illumination), which reduces the shadows that would otherwise obscure the deeper parts of the access cavity [19,27]. Two filters are commonly used. An orange or yellow filter prevents the premature polymerization of light-cured resin materials during placement, and a green filter reduces the red component of the field, which increases the contrast between bleeding and non-bleeding tissue and is useful during surgery [19,27]. Because the light source is very bright, the consensus recommends eye protection for the patient and the assistant and advises against prolonged continuous use [19].

Positioning and Four-Handed Dentistry

The consensus recommends establishing the operator’s position first, then the patient’s position, and finally the working distance and initial focus of the microscope. The operator usually sits at the 12 o’clock position, with the 9 o’clock position as an alternative, and moves within this range as required. Rubber dam isolation and four-handed dentistry are regarded as prerequisites, and the dental assistant requires specific training in four-handed microscope work [19]. Operator height also matters. In one study, mandibular molars took longer to bring into view than maxillary teeth in all operator height groups, and shorter operators had to adopt a strained posture to view mandibular teeth; this difficulty was reduced by angled binoculars or a 200 mm objective lens [35]. In the 2008 survey, shorter endodontists used the microscope more often than taller endodontists [20].

Documentation and Teaching

Integrated cameras allow images and videos to be recorded during treatment for clinical records, patient communication, teaching, and remote consultation [19]. Photomicrography requires careful adjustment of both the microscope and the camera and involves its own learning curve [42]. Video transmission has been proposed as a tool for distance education in endodontics [43].

Non-surgical root canal treatment

Role of the Microscope

In non-surgical root canal treatment, the DOM is used mainly to improve visualization during access cavity preparation, to locate canal orifices, to identify cracks and other defects, and to verify each step of treatment [44]. Contracted access cavity designs conserve tooth structure but restrict the view of the pulp chamber floor. A systematic review with meta-analysis [45] and a micro-CT study that assessed canal detection in maxillary molars sequentially without magnification, under the microscope, and after ultrasonic troughing suggest that magnification compensates for part, but not all, of this visual limitation [46].

Missed Canals and Treatment Failure

Canals that are not located remain uncleaned and unfilled and can harbor microorganisms and necrotic tissue that sustain periapical inflammation [2,8]. In a cone-beam computed tomography (CBCT) study of endodontically treated premolars and molars, teeth with an untreated canal were significantly more likely to show apical periodontitis than teeth in which all canals had been treated [11]. A CBCT study of 1,024 endodontically treated posterior teeth in a Chinese subpopulation similarly found a significant association between missed canals and periapical lesions [12]. Failure to locate the second mesiobuccal (MB2) canal of maxillary molars is one of the most common reasons for persistent disease in these teeth [8], which explains why MB2 detection has become the most frequently used measure of the value of magnification.

Detection of the MB2 Canal and Other Canals

In an in vivo study of 312 maxillary first and second molars treated by endodontists, Buhrley et al. located the MB2 canal in 18.2% of teeth treated without magnification, 55.3% of teeth treated with loupes, and 57.4% of teeth treated with the microscope; in maxillary first molars, the corresponding values were 17.2%, 62.5%, and 71.1% [40]. The difference between the microscope and loupes was not statistically significant, which indicates that the largest gain occurs when any form of magnification is used.

Ultrasonic troughing provides an additional benefit. Alaçam et al. examined 100 extracted maxillary first molars sequentially and detected the MB2 canal in 62% of teeth without the microscope, in 67% with the microscope, and in 74% with the microscope combined with ultrasonic troughing; sectioning showed a true prevalence of 82% [47]. In a two-factor experimental study by Adiguzel and Ozeken, detection was lowest with a videomicroscope without troughing (40.4%) and highest with the DOM combined with troughing (88.5%); troughing alone and magnification alone achieved 57.7% and 67.3%, respectively, and both magnification (odds ratio [OR] = 3.90) and troughing (OR = 2.54) were independent predictors of detection [48]. A three-stage ex vivo study of 208 maxillary molars examined by undergraduate students [49] and an in vivo study by Sujith et al., in which the microscope revealed the MB2 canal in 21 teeth that had been missed by the naked eye and troughing revealed a further nine canals [50], showed the same sequential pattern.

The benefit is not limited to the MB2 canal. In mandibular single-rooted teeth, Çulha et al. reported a sensitivity for detecting a lingual canal of 0.40 with direct visual inspection, 0.59 with ×6 loupes, and 0.68 with the DOM; both forms of magnification were significantly better than direct vision, with no significant difference between them [51]. Magnification and CBCT are complementary rather than interchangeable. Studies comparing clinical inspection under magnification with CBCT treat them as separate diagnostic methods [52,53], and the accuracy of CBCT in detecting the MB2 canal is reduced by artifacts from existing root fillings [54]. These reports illustrate the clinical technique but, as single cases, cannot establish its effect on outcomes (Figure 2).

**Figure 2:**
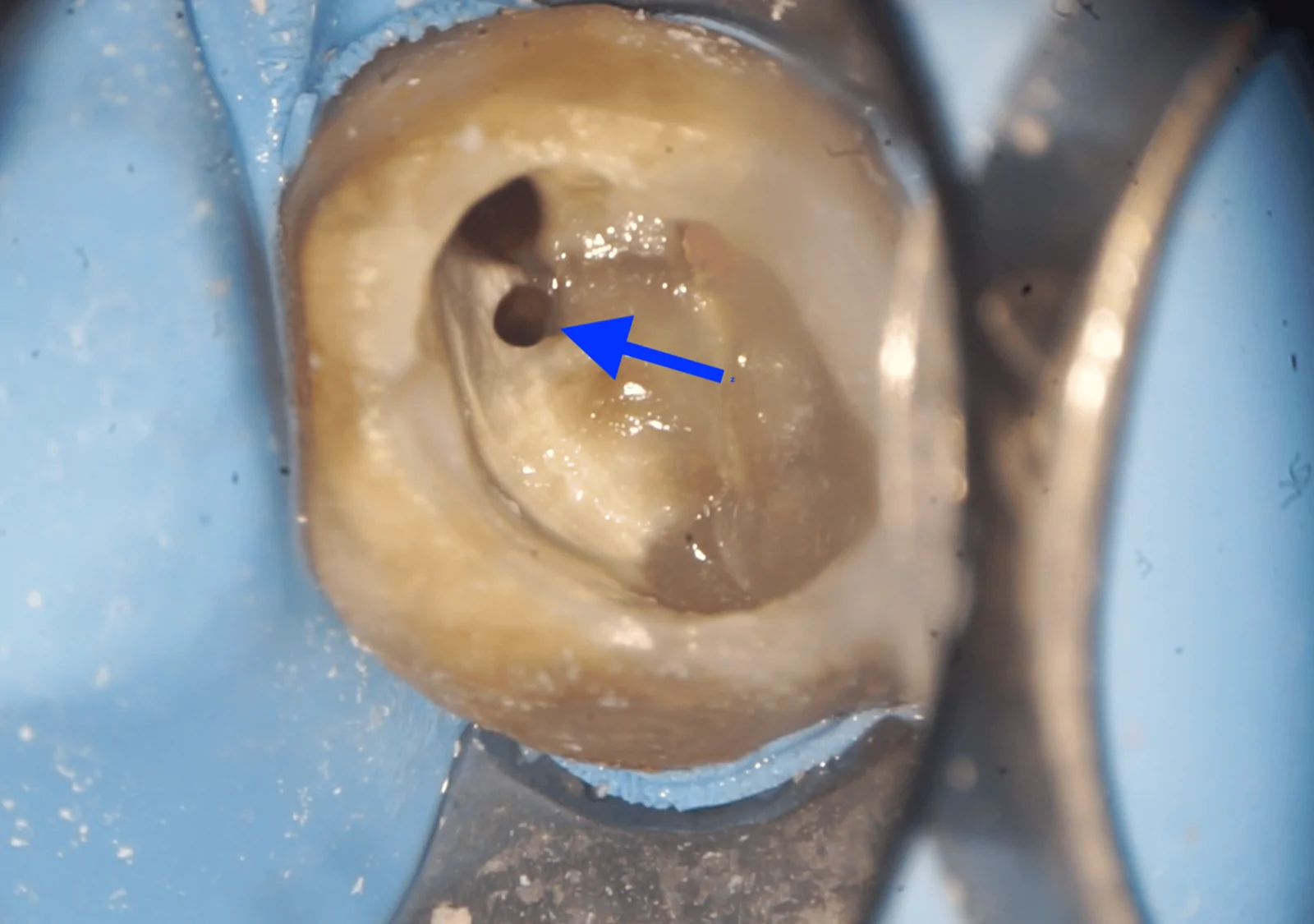
Identification of complex root canal anatomy under high-power magnification The blue arrow demonstrates the precise location of the MB2 canal in a maxillary molar. This image illustrates how the DOM assists clinicians in negotiating challenging anatomical variations and locating orifices that might be missed with the naked eye. Intraoral view captured by the author using their own clinical DOM during a case treatment. MB2: Second mesiobuccal; DOM: Dental operating microscope

Complex Root Canal Anatomy

The DOM is also used to identify and manage anatomical variations such as C-shaped canal systems and additional roots. In such cases, CBCT is typically used to identify the variation before treatment, while the microscope is used to follow isthmuses and fins, to locate orifices that are displaced from their expected position, and to remove dentine selectively with ultrasonic tips. This combined approach has been described in case reports of the retreatment of a mandibular second molar with a C-shaped canal system [55], the treatment of a maxillary first molar with two separate palatal roots [9], and the location of three calcified canals in a mandibular first molar with a radix entomolaris and five canals [10].

Outcome Evidence

Improved detection is a surrogate outcome. The most direct outcome data in non-surgical treatment come from a retrospective case-control study of teeth presenting for retreatment by Khalighinejad et al. [56]. The mesiobuccal roots of maxillary molars were more likely to show periapical radiolucency when the initial treatment had been performed without a microscope (OR = 3.1). A missed MB2 canal was significantly associated with periapical radiolucency in teeth initially treated without a microscope (OR = 5.1) but not in teeth treated with a microscope [56]. These findings indicate an association, not a causal effect. The microscope itself does not influence periapical inflammation; the most plausible explanation is that it increases the likelihood that all canals are located and disinfected, which is consistent with the association between untreated canals and apical periodontitis described above [11,12]. Because of the retrospective design, differences in operator experience and case selection cannot be excluded.

Calcified Canals

Calcified canals were among the first non-surgical indications for the DOM [29]. Under magnification, color differences between the pulp chamber floor, reparative dentine, and the surrounding dentine, and the subtle outline of a calcified orifice, can be followed while dentine is removed selectively with ultrasonic tips, which reduces the risk of perforation [27,57]. A clinical study of elderly patients treated in a practice limited to endodontics reported the accessibility of calcified canals, the time required, and the outcomes after three years [58]. A systematic review of case reports concluded that the combined use of the DOM and ultrasonic tips was reported to improve outcomes in several cases and proposed an updated decision-making algorithm for pulp canal obliteration [57]. The CBCT-aided microscopic and ultrasonic treatment has also been described for canals calcified in the coronal or middle third [59], and a case report of two calcified mandibular central incisors followed for up to six years illustrates the integration of CBCT, the DOM, and ultrasonic instruments [60]. As with complex anatomy, this evidence is largely descriptive (Figure 3).

**Figure 3:**
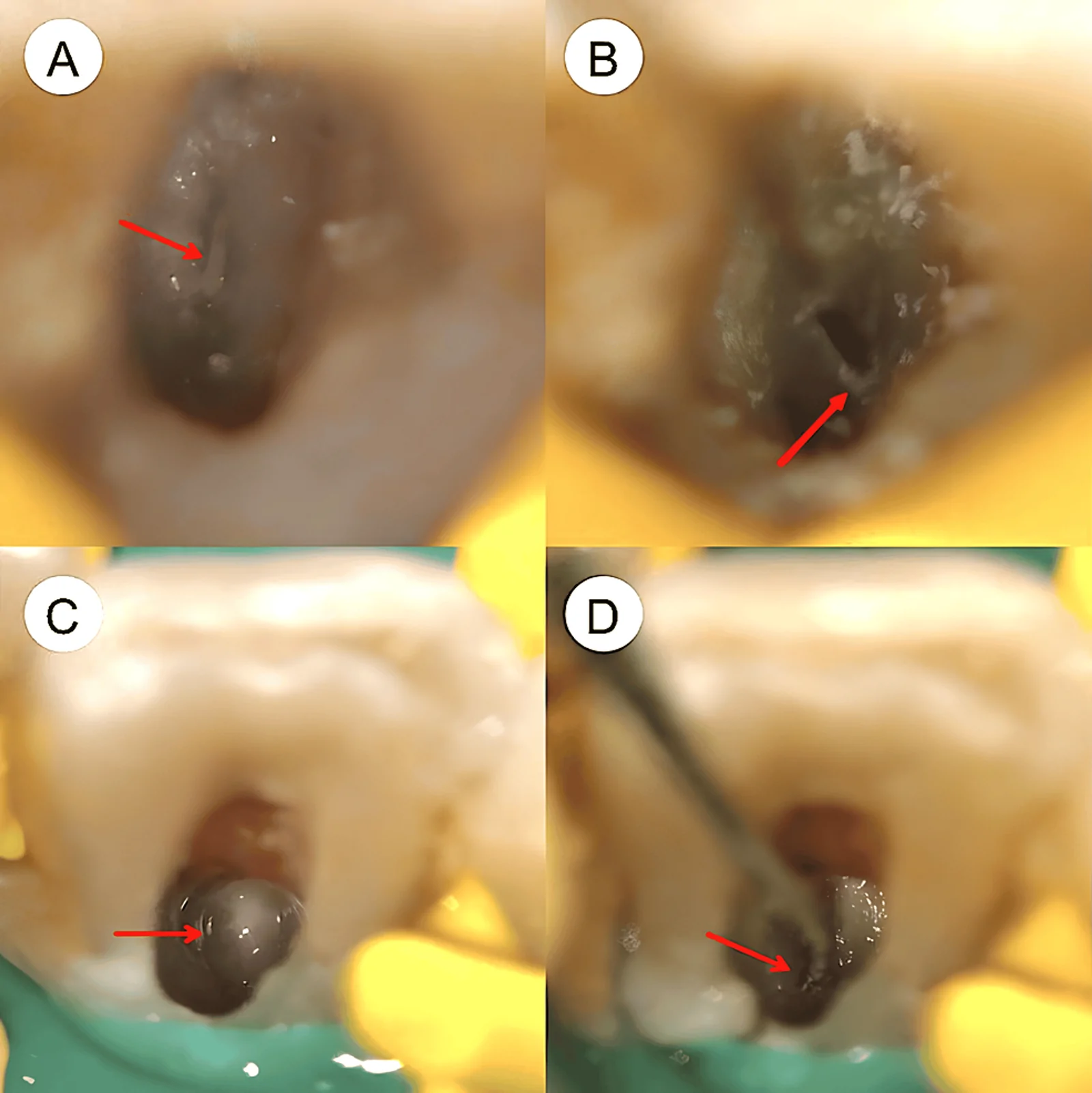
Microscope-assisted management of calcified root canals Clinical views demonstrating the negotiation of obscured anatomy under magnification [60]. A: Red arrow highlights severe calcification on the pulpal floor of tooth #31; B: Successfully identified root canal orifice (red arrow) in tooth #31; C: Similar presentation of calcification (red arrow) within the pulp chamber of tooth #41; D: Successfully negotiated canal orifice (red arrow) in tooth #41 following microscope-assisted exploration

Non-surgical retreatment and management of procedural complications

Non-surgical retreatment involves the removal of existing root filling materials, the location of previously untreated canals, and the management of procedural errors; the case of a C-shaped mandibular molar described above is an example [55]. The ultrasonic technique used under the microscope removes separated instruments more predictably than less controlled approaches, and the time needed for removal depends mainly on canal curvature and fragment length [61]. A 10-year retrospective study described the pattern of instrument separation and the factors associated with successful retrieval [62].

For perforation repair, two outcome studies, one of 26 teeth followed for 12 to 65 months and one of 90 teeth followed for one to 10 years, used mineral trioxide aggregate placed under the DOM [63,64]. In both studies, the microscope was part of the treatment protocol rather than the variable being tested, and recent reviews of perforation management use the same approach [65]. One micro-CT study did compare magnification levels directly and evaluated the obturation quality of furcation perforation repairs performed at ×4 and ×10 [66]. In invasive or external cervical resorption, the margin of the defect must be followed and the resorptive tissue removed under direct vision, which makes high magnification particularly useful [67,68].

In a clinical series of 345 teeth with 546 canals that could not be managed with conventional methods because of calcification, separated instruments, missed canals, or perforations, treatment under the DOM with ultrasonic instruments was successful in 74.4% of canals and 71.3% of teeth [69]. Although the study had no comparison group, it suggests that the microscope can allow some teeth that would otherwise require surgery or extraction to be treated non-surgically. When non-surgical retreatment is not feasible or has failed, endodontic microsurgery is the main alternative, and the outcomes of the two approaches have been compared in a meta-analysis [70].

Vital pulp therapy

Contemporary vital pulp therapy depends on intraoperative assessment of the exposed pulp. After caries removal, the clinician evaluates the color, consistency, and integrity of the pulp tissue and whether hemostasis can be achieved after irrigation with sodium hypochlorite; these findings guide the choice between direct pulp capping, partial pulpotomy, and full pulpotomy [6,71]. The DOM allows these features to be assessed in more detail, supports the complete removal of infected dentine around the exposure, and facilitates the precise placement of calcium silicate materials. A randomized clinical trial comparing direct pulp capping and partial pulpotomy in mature teeth with reversible pulpitis performed this assessment under magnification [72], and a review of pulpotomy outcomes identified intraoperative evaluation of pulp status as a determinant of success [73]. However, no study has isolated the effect of the microscope itself on the outcomes of vital pulp therapy.

Regenerative endodontic procedures (REPs)

Regenerative endodontic procedures are indicated mainly for immature permanent teeth with pulp necrosis. They aim to promote continued root development through the formation of vital tissue within the canal space [7,74]. Several steps of the procedure require precise visual control. Because mechanical instrumentation is minimal to preserve the thin dentinal walls, disinfection relies on irrigation and intracanal medicaments, and magnification helps to identify residual necrotic tissue and medicament in the wide canal [7]. At the second appointment, bleeding is induced from the periapical tissues to fill the canal to a predetermined level, a collagen matrix is placed over the blood clot, and a calcium silicate cement and a coronal seal are then applied [7,74]. Under the microscope, the level of the blood clot, the position of the matrix, and the thickness and adaptation of the calcium silicate layer can be verified directly, and potentially discoloring materials can be kept below the cervical area. The procedure has also been applied to complicated cases, such as traumatized immature teeth with severe external root resorption and root perforation [75]. As with vital pulp therapy, no studies have compared REPs performed with and without a microscope, and its use in these procedures is supported by technical rationale rather than by comparative outcome data.

Endodontic microsurgery

Setzer et al. conducted a meta-analysis of root-end surgery that compared contemporary root-end surgery, in which microinstruments and modern materials were used with loupes or without magnification, with endodontic microsurgery, in which the same techniques were combined with high-power magnification from a surgical microscope or an endoscope [76]. The weighted pooled success rate was 88% (95% confidence interval (CI) 0.8455-0.9164) for contemporary root-end surgery and 94% (95% CI 0.8889-0.9816) for endodontic microsurgery. The difference was significant (p < 0.0005), with a relative risk ratio of 1.07 [76]. The benefit was significant for molars but not for premolars or anterior teeth, possibly because access and root anatomy are more demanding in molars.

In a retrospective study by Tsesis et al., the modern technique, which combined minimal or no root-end bevel, ultrasonic root-end preparation, and the microscope, had a significantly better outcome than the traditional technique, which used a 45-degree bevel and bur preparation [77]. In contrast, Taschieri et al. compared loupes and the surgical microscope within an otherwise identical microsurgical protocol [41]. The success rates were 91.7% with loupes and 91.4% with the microscope at one year, and 90.5% and 93.3%, respectively, at four years; the differences were not statistically significant [41]. Taken together, these studies suggest that much of the improvement associated with modern endodontic surgery comes from the protocol as a whole, including ultrasonic root-end preparation and biocompatible root-end filling materials, rather than from the level of magnification alone.

Several studies have examined whether early healing after microsurgery is maintained. When cases judged to be healed one year after apical microsurgery were re-examined five to seven years later, 54 of 59 roots (91.5%) remained healed [78]. A cohort of 172 cases classified as successful at short-term follow-up and followed for up to 10 years showed similar results [79], and a comparison of one-year and long-term assessments confirmed that early findings largely predict long-term outcomes [80]. These findings are supported by a meta-analysis of long-term prognosis [81].

Magnified inspection has also helped to identify prognostic factors. Root dentinal defects detected on the resected root surface with a microscope and a fiber-optic probe were associated with poorer short- and long-term outcomes [82]. A systematic review with meta-analysis identified lesion size of 5 mm or less as an important variable among hard tissue defects [83], and periapical and marginal bone deficiency [84], combined endodontic-periodontal lesions [85], and periodontal attachment loss [86] have each been associated with poorer outcomes. A clinical study that inspected root surfaces under the microscope before and after ultrasonic root-end preparation addressed the concern that the preparation itself might cause damage, with reassuring results [87]. The outcomes of repeat microsurgery after failed primary microsurgery have been evaluated with propensity score-matched survival analysis [88]. Current reviews consider the microscope, CBCT planning, piezoelectric osteotomy, and biocompatible root-end filling materials as components of a single integrated protocol [89,90]. The microscope also allows less common surgical approaches; for example, a palatal microsurgical approach was used to manage a maxillary lateral incisor with a palatal sinus tract, with a five-year follow-up (Figure 4) [91].

**Figure 4:**
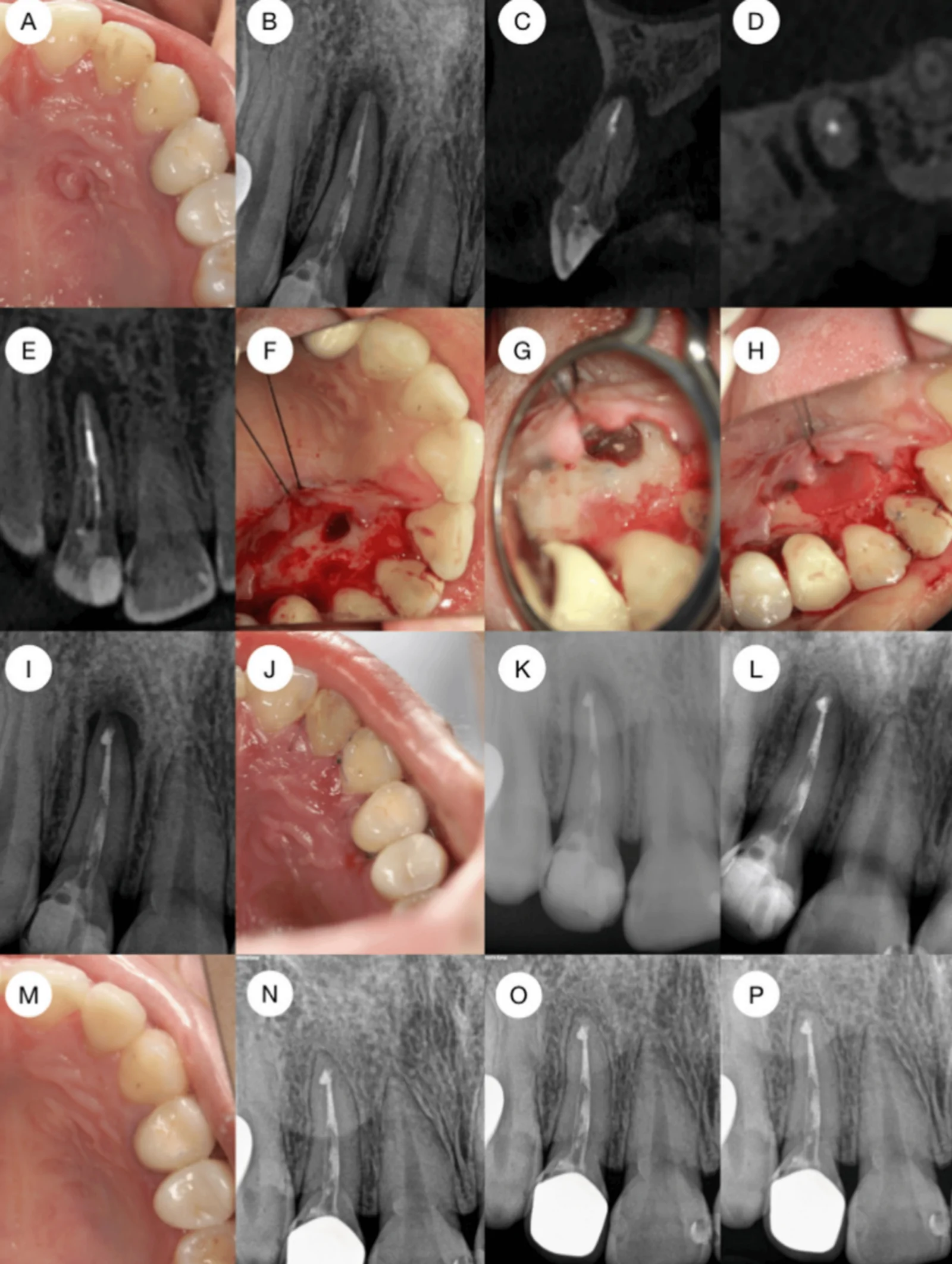
Comprehensive management of a maxillary lateral incisor via a palatal microsurgical approach Clinical and radiographic sequence of palatal endodontic microsurgery with a five-year follow-up [91]. A: Preoperative clinical view demonstrating a palatal mucosal swelling; B: Initial periapical radiograph indicating an apical radiolucency associated with tooth #12; C-E: Preoperative CBCT slices in the sagittal (C), axial (D), and coronal (E) planes confirming the extent of the periapical lesion; F: Clinical photograph of the elevated and sutured triangular palatal mucoperiosteal flap; G: Surgical site following root-end resection, preparation, and retrofilling with a bioceramic materia; H: Placement of a resorbable barrier membrane over the bony defect; I: Immediate postoperative radiograph; J: One-week follow-up showing early soft tissue healing and resolution of the sinus tract; K-L: Radiographic evaluations at three (K) and six (L) months evidencing progressive osseous healing; M: One-year clinical follow-up displaying excellent palatal mucosal tissue recovery without visible scarring; N: One-year radiograph showing complete periapical resolution and re-establishment of the lamina dura; O-P: Long-term radiographic stability at two years (O) and five years (P) post-surgery CBCT: Cone-beam computed tomography

Ergonomics and education

The DOM promotes an upright working posture because the operator must look through fixed eyepieces. In addition to the studies discussed in the Introduction [16-18], a crossover study of endodontic residents found that a tailored ergonomics lecture reduced postural risk under all magnification conditions [92], and an experiential microscope-based ergonomics curriculum evaluated with Kirkpatrick’s model produced measurable improvements [93]. These findings indicate that the microscope makes a better posture possible, but training is needed to achieve it.

The DOM has been used as an educational tool since the 1990s [94]. Undergraduate students trained with the microscope improved significantly in access cavity preparation and identified canals more accurately than students in comparison groups when instructional content and time were kept constant [95], and a micro-CT study of undergraduate access cavities reached similar conclusions [96]. A systematic review of magnification and undergraduate cavity preparation, however, took a more cautious view of the strength of this evidence [97]. The stage at which magnification is taught appears to influence whether graduates use it later in practice [98]. Working at high magnification requires precise bimanual coordination under altered visual and motor conditions. The microsurgical literature describes the acquisition of this skill as requiring structured, deliberate practice [99], together with non-technical skills such as situation awareness, decision-making, communication, and teamwork [100].

Use in other dental disciplines

Outside endodontics, the evidence is mixed. In restorative dentistry, a scoping review concluded that magnification improves preparation performance compared with no magnification, but that the higher magnification of the microscope offers no clear advantage over loupes [101]. This question has also been examined in a randomized controlled trial of class II composite restorations placed with the naked eye, ×3 and ×5 loupes, or an ×8.5 DOM [102], and in a micro-CT study of marginal gaps in computer-aided design and computer-aided manufacturing (CAD-CAM) crown preparations [103]; arguments for the role of the microscope in minimally invasive restorative dentistry have also been presented [104,105]. In periodontology and implant dentistry, the evidence is more favorable: a systematic review summarized current clinical applications [106], a meta-analysis found microsurgery superior to conventional surgery for root coverage [107], and a randomized controlled trial compared the microscope with loupes for alveolar ridge preservation [108]. The development of periodontal microsurgery has followed a course similar to that of endodontic microsurgery [109].

Limitations and contraindications

The main limitation is the quality of the evidence. Two Cochrane reviews of magnification devices in endodontic therapy, published in 2009 and updated in 2015, found no study that met the inclusion criteria [21,22], and a systematic review of endodontic therapy with magnification devices reached a similarly cautious conclusion [110]. The case for the microscope therefore rests on cohort, case-control, ex vivo, and meta-analytic evidence rather than on randomized trials, and this should be acknowledged when the DOM is described as a standard of care.

The practical limitations are easier to quantify. In the comparison of the DOM and the videoscope, procedures took on average 1.9 and 2.8 times longer, respectively, than with loupes, and students took 1.3 times longer than residents regardless of the device used; at the highest magnification setting, the depth of field was only 6 mm [39]. Cost and a lack of trained staff were the main barriers reported by dental school teaching leads [33]. The bright light requires eye protection for the patient and the assistant, and because the operator’s eyes remain at the eyepieces, communication with the team is restricted, and four-handed dentistry becomes essential [19].

There are no absolute contraindications, but several situations limit the usefulness of the microscope. Limited mouth opening, an unfavorable tooth position, or a patient who cannot maintain the required head position may prevent a clear line of sight, because the optical axis of the microscope must be aligned with the operating field [19,35]. Patients who cannot tolerate long appointments may be poorly served by a technique that increases treatment time, and an operator without adequate training may work more slowly at high magnification without working more accurately [39,99]. The consensus states that the role of the DOM should not be exaggerated, that its indications should not be extended indefinitely, and that it does not need to be used throughout an entire treatment [19].

Finally, the microscope shows only visible surfaces. A vertical root fracture that does not extend to an accessible surface cannot be detected with it, and the in vivo diagnostic accuracy of radiographic methods for this condition is also limited [111]. Magnification and 3D imaging therefore answer different clinical questions, and neither can replace the other.

Current and emerging directions

Several technologies are being combined with the microscope. A systematic review and meta-analysis assessed the accuracy of static computer-aided navigation in locating the root apex during endodontic microsurgery [112], and CBCT combined with 3D printing has been used to guide minimally invasive microsurgery [113]. Dynamic navigation has been compared with a 3D microscope for the retrieval of separated files [114] and with freehand preparation for canal location in extracted molars [115], and it has been combined with the DOM to manage pulp canal obliteration [116]. Robotic assistance has been reported for locating a calcified canal [117] and for autonomous osteotomy and root-end resection during microsurgery of mandibular molars [118]. In these reports, navigation or robotic systems provide accuracy of the drilling path, whereas the microscope provides visual confirmation. The videoscope and the endoscope remain the closest alternatives: the videoscope is not restricted to a fixed optical axis but has lower resolution and a shallower depth of field than the microscope, and its accuracy did not differ significantly from that of loupes [39]. The literature on endodontic endoscopy is older and smaller [119], and neither device has replaced the microscope in routine use.

## Conclusions

The dental operating microscope is a valuable tool in endodontics, but its value differs between procedures. Its clearest benefit is improved visualization, which helps clinicians to locate canals, manage calcified and complex anatomy, retrieve separated instruments, repair perforations, and inspect resected root surfaces. In endodontic microsurgery, high-power magnification is associated with higher success rates, particularly in molars, although much of the benefit reflects the modern microsurgical protocol as a whole. In non-surgical treatment, vital pulp therapy, and regenerative endodontic procedures, the microscope improves precision and control, but its independent effect on long-term outcomes has not been established. Its use should therefore be guided by the size of the clinical target and the consequences of not seeing it, rather than being applied routinely to every step of every procedure. The additional treatment time, cost, and training required should also be considered. Randomized controlled trials and prospective studies with patient-centered outcomes are needed to confirm whether the diagnostic advantages of the microscope lead to better healing and tooth survival.
